# Structure-filtered search of enzyme variants

**DOI:** 10.1016/j.csbj.2025.09.039

**Published:** 2025-10-01

**Authors:** Linda Porri, Sandra Castillo, Paula Jouhten

**Affiliations:** a Department of Bioproducts and Biosystems, Aalto University, Espoo, Finland; b VTT Technical Research Centre of Finland Ltd, Espoo, Finland

**Keywords:** AlphaFold, Enzyme variant, Fungi, Non-ribosomal peptides, PSI-BLAST

## Abstract

Performance characteristics of a heterologously expressed enzyme may be critical for an application but they arise from the complex sequence, structure, function-dependency. Since the sequence-structure-function insights are limited, there is typically a need to screen a large number of sequence-similar enzyme variants. The scale of screening needed hinders the industrial strain development by laboratories smaller than biofoundries.

We present an *in silico* method for refining an enzyme candidate set for experimental screening by structural similarity filtering. The method searches naturally occurring, non-redundant but functionally equivalent orthologs by combining typical homologous sequence search with filtering by structural similarity using AlphaFold-predicted protein structure models. We demonstrated the method for finding enzyme candidates similar to non-ribosomal peptide synthetases aspergillic acid synthetase (asaC) from *Aspergillus flavus* and chrysogine synthetase (chyA) from *Penicillium rubens*. The sequence similarity searches alone yielded tens of thousands of candidates of each kind. However, filtering by global structural similarity and the assessment of active site residues effectively narrowed down the enzyme candidates to 24 similar to asaC and one similar to chyA. Thus, filtering by structural similarity can efficiently refine enzyme candidate sets reducing the experimental screening effort and accelerating the development of strains for applications.

## Graphical Abstract

**Figure:**
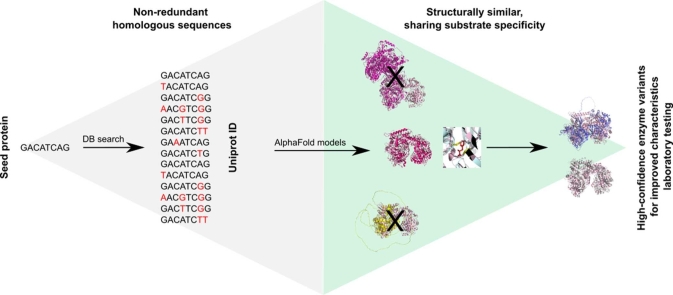

## 1 Introduction

Living cells can produce a wide range of commercially valuable compounds such as secondary metabolites of microbes and plants. However, the native host organisms may not be cultivable in industrial scale and the synthesis of the compounds may occur in low quantities and only in very specific conditions. To achieve high production levels in industrial-scale, heterologous expression of enzymes and pathways in industrially cultivable microbial hosts have proven effective [1]. Yet, an enzyme from a known native host organism often turns out inefficient when expressed in the production host. Therefore, improved enzyme characteristics are commonly sought after, not only for feasible but also for economically competitive biotechnological production. Enzyme characteristics can be engineered rationally by introducing targeted mutations to the amino acid sequence based on prior knowledge, or by randomized mutagenesis and high-throughput screening [2]. Both approaches suffer from the huge combinatorial challenge arising from the exponentially by length growing number of possible sequence variants whose effects on the enzyme function depend on complex interactions between the residues. Unfortunately, detailed understanding of how the sequence, structure, and function correlate is limited, and rational or random variants are too numerous to be exhaustively screened [3], [4]. Thus, the screening is commonly focused on naturally occurring enzyme variants, in particular orthologs with shared primary function. However, bioinformatic searches for orthologs of a specific enzyme in sequence resources can yield too large to screen, redundant sets, and algorithms like PSI-BLAST may inadvertently include non-homologous sequences, complicating the identification of true orthologs [5].

Here, we present a method utilizing AlphaFold-predicted protein structure models to filter the sequence-similar sets of enzyme candidates for extracting orthologs that are globally and in active site structurally similar to the seed enzyme. The structurally similar enzymes are expected to most likely also share the primary function. We demonstrate the method by searching non-ribosomal peptide synthetase (NRPS) candidates, specifically the variants of aspergillic acid synthetase (asaC) from *Aspergillus flavus* and chrysogine synthetase (chyA) from *Penicillium rubens*. Penicillin and cyclosporine are examples of non-ribosomal peptides (NRPs) that are already biotechnologically produced for commercial use. NRPs are synthesized from a variety of possible substrates, including but not limited to proteinogenic amino acids, fatty acids, non-proteinogenic amino acids or carbohydrates [6]. While an NRPS consists of a variable number of modules and domains, the adenylation (A) domain is the main mediator of the substrate specificity and directs the incorporation of a substrate into the growing peptide chain [7], [8]. However, the activity of the A domain and the specificity of the NRPS final product may be influenced by the C domain [9], [10]. Interestingly, the A and C domains may not share the evolutionary history [11] and sequence alignments of A domains have not shown notable correlation with NRPS function [12]. Thus, the sequence searches of NRPSs should be performed for complete genes beyond only the A domain. A holistic sequence similarity search with asaC and chyA enzyme sequences as seeds retrieved tens of thousands of putative orthologs from four public sequences databases. However, after filtering by structural similarity including an assessment of active site residues, only 24 candidates for asaC and a single one for chyA were found to likely share their primary functions. Thus, chyA variants should be searched in further sequence resources such as metagenomes, but asaC variants represent a promising set for experimental screening even in small-scale laboratories. These outcomes demonstrate the potential of structure-filtered search of enzyme candidates.

## 2 Material and methods

### 2.1 Protein sequence-based search for naturally occurring enzyme variants

**Fig. 1:**
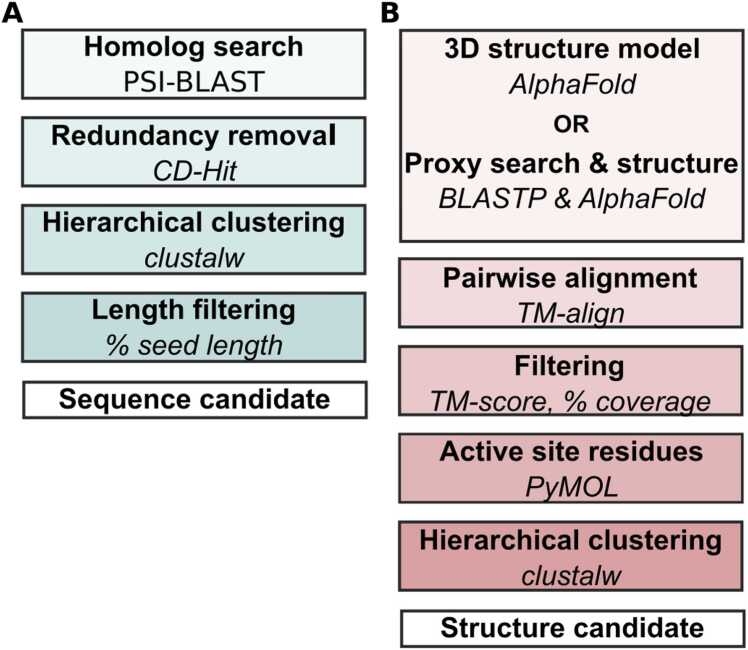
Workflow to identify the most likely functionally equivalent enzyme candidates. a) Search and prioritization yielding sequence similarity -based candidates, b) filtering by structural similarity and assessing the similarity of the active site residue composition yielding high-confidence candidates for experimental screening of enzyme performance characteristics.

Seed sequences in FASTA format were downloaded from Uniprot (https://www.uniprot.org/; asaC: B8N0E8, chyA: B6HLP9). Then, the seed sequences were searched against the protein sequence databases nr, SwissProt, UniProt, TrREMBL using PSI-BLAST [13] with a BLASTP e-value threshold of 10, a PSI-BLAST inclusion e-value threshold of 0.001, and 4 PSI-BLAST iterations. A non-redundant set of sequences was obtained by clustering with ≥ 99 % identity using CD-Hit [14]. The evolutionary relationships between candidate sequences were explored using ClustalW with default settings [15] and visualized using the Interactive Tree of Life web tool [16]. The non-redundant sequences were filtered based on sequence length similarity to the seed protein assuming that sequences significantly longer or shorter than the seed protein sequence would either possess additional unrelated functions or lack the required primary activity (Fig. 1 A).

### 2.2 Structural similarity evaluation

AlphaFold Monomer V2.0 predicted protein structure models were downloaded from the AlphaFold Protein Structure Database (https://alphafold.ebi.ac.uk/). The AlphaFold protein structure database did not contain protein structure models for all candidate sequences, and some had no UniProtID to query the database with. Hence, to estimate indirectly the global structural similarity between a candidate and a seed, we searched for proxy proteins in the SwissProt database. By using a ≥ 95 % sequence identity threshold for the BLASTP searches, a proxy was found for each of the candidate sequences lacking an AlphaFold protein structure model. Candidate and seed structures were aligned pairwise using TM-align [17]. Structural similarity was defined by a TM-score between 0.5 and 1.0, and seed coverage of the variant to seed structure of ≥ 70 %, calculated based on the length of the aligned sequences (Fig. 1 B).

### 2.3 Identification of active site residues

The crystal structure of Gramicidin Synthase (GrsA) in complex with phenylalanine (PBD ID: 1AMU) was retrieved from the RCSB Protein Data Bank (https://www.rcsb.org/). The structure of a seed protein was superimposed onto GrsA using PyMOL Molecular Graphics System Version 3.0 (Schrödinger, LLC). Amino acids within 5 Å distance of phenylalanine in GrsA were selected, polar interactions with the protein backbone identified, and mapped to the corresponding amino acids in the seed model. Active site residues across all enzyme candidates were identified by local pairwise sequence alignment to the seed using Bio.pairwise2/Biopython [18] and the following scoring scheme: +2 for identical characters, -1 for mismatches, -0.5 for gap openings, -0.1 for gap extensions (Fig. 1 B).

## 3 Results and discussion

### 3.1 Sequence-based search yielded numerous potential candidates

**Fig. 2:**
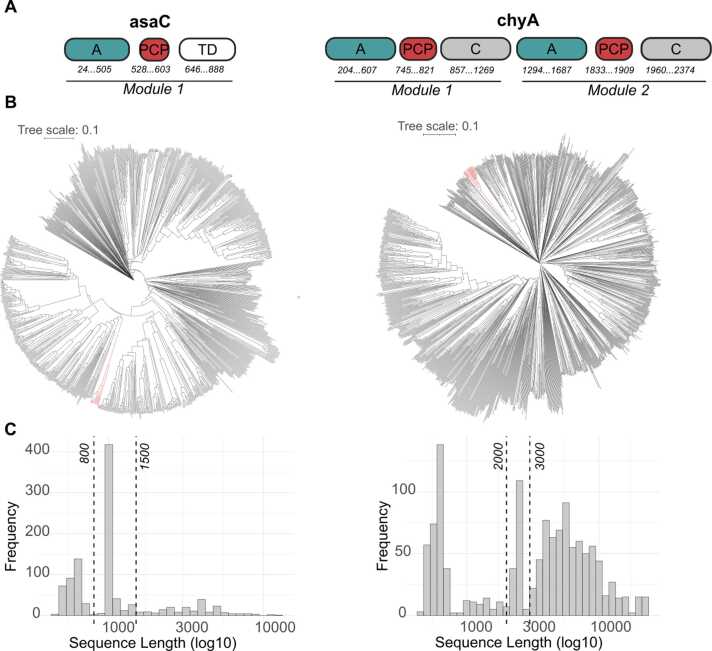
Search for sequences similar to asaC and chyA. a) NRPS module and domain structure. b) Phylogenetic trees of asaC and chyA (branch marked red) and orthologous sequences. c) Candidates meeting the sequence length criteria between the vertical lines. (PCP: peptidyl carrier protein domain; A: adenylation domain; C: condensation domain; TD: terminal reductase domain).

The NRPSs asaC and chyA produce aspergillic acid, an antibiotic that forms an orange pigment when complexed with iron, and a yellow pigment chrysogine, respectively [19], [20], [21]. These compounds have not yet been heterologously produced, and the synthesizing enzyme complexes have relatively simple architectures compared to many other NRPSs (Fig. 2 A). A sequence-similarity based search in public databases identified a total of 15405 sequences similar to asaC and 76367 to chyA. Among these, 1027 and 1271 were non-redundant sequences similar to asaC and chyA, respectively. Clustering of the non-redundant sequences revealed low branch distances and a dispersed distribution of NRPS/NRPS-like proteins, indicating that these sets contained relevant sequences for further characterization (Fig. 2 B). Sequence length filters (800–1500 aa for asaC (1021 aa); 2000–3000 aa for chyA (2382 aa)) were applied to enrich for enzymes with global architectures similar to the seed proteins, yielding 503 asaC and 158 chyA candidates, respectively (Fig. 2 C). Since the enzyme sequences could be fragmented in metagenomic sequence resources, sequence length filters should be adjusted depending on the data source.

### 3.2 Filtering by structural similarity identified high-promise enzyme variants

Next, we retrieved AlphaFold protein structure models for structural filtering of the candidates. AlphaFold protein structure models were readily available for 211 out of the 503 candidates sequence-similar to asaC. For 292 asaC -like candidates the AlphaFold database did not contain protein structure models. Therefore, AlphaFold protein structure models of proxy proteins having ≥ 95 % sequence-similarity to the candidates were retrieved. At the ≥ 95 % sequence similarity threshold for a proxy protein it was possible to acquire a protein structure model for each candidate. Although the ≥ 95 % sequence-similar proteins cannot not be expected to have identical structures to the corresponding candidates, the *de novo* prediction of the lacking AlphaFold protein structure models would have had a notable carbon-footprint. Though less for shorter proteins like asaC, the carbon-footprint of predicting an AlphaFold protein structure model for a large protein like chyA could be up to ∼few kg CO₂e [22]. Thus, despite the uncertainty increase we considered the models of proxy proteins suited for filtering candidates by the global structural similarity to the seeds. The global structural similarity was inferred by performing a pairwise structural alignment of the AlphaFold protein structure models representing the candidates and the model of asaC, 14 out of the 211 variants with directly available AlphaFold protein structure models were predicted to be structurally similar to asaC. In addition, 38 out of the 292 candidates represented with AlphaFold protein structure models of proxy proteins were predicted to be structurally similar to asaC (Suppl. Table 1).

**Fig. 3:**
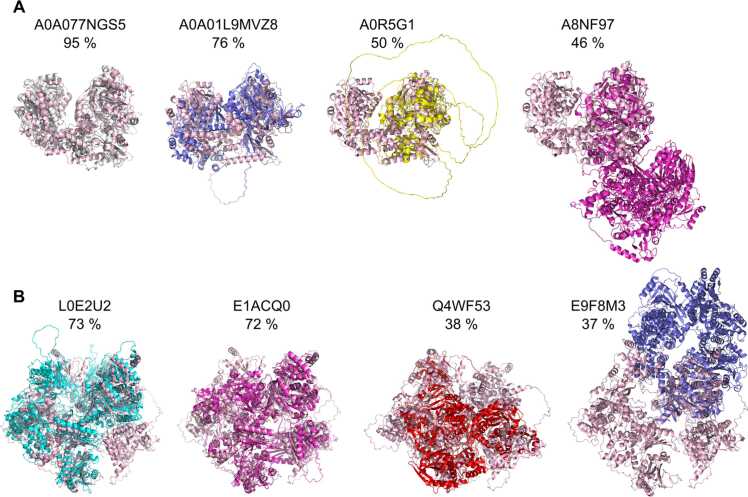
Exemplary pairwise alignments of enzyme variants (various colors) with different sequence coverages to asaC or chyA (light pink). a) A0A077NGS5 and A0A01L9MVZ8, but not A0R5G1 and A8NF97, represented candidates likely structurally similar to asaC with high sequence coverage (≥70 %), as well as b) L0E2U2 and E1ACQ0, but not Q4WF53 and E9F8M3 to chyA.

In case of chyA, for 37 candidates similar to chyA AlphaFold protein structure models were readily available, while for 121 candidates the models of ≥ 95 % sequence-similar proxy proteins were retrieved. The pairwise structural alignment with the chyA AlphaFold protein structure model revealed that only four candidates out of the total 158 candidates were predicted to be structurally similar to chyA (Suppl. Table 2). Following from the predicted structural similarities, a seed sequence coverage of ≥ 70 % appeared to enrich for structurally similar variants of asaC or chyA (Fig. 3).

**Fig. 4:**
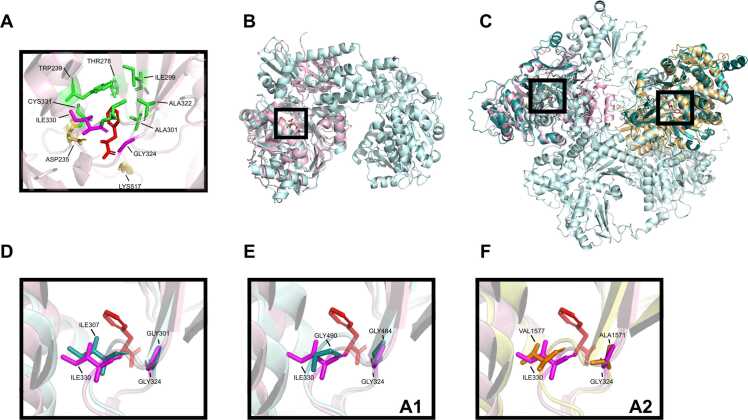
Active site residue identification in enzyme candidates. a) The Stachelhaus-code residues in the substrate binding pocket (green), the residues mediating electrostatic interactions (yellow), as well as the active site residues our method identified (magenta) in relation to the phenylalanine substrate (red). Structural alignment of b) the asaC (B8N0E8; lightblue) or c) the chyA AlphaFold structure model (B6HLP9; lightblue) to the GrsA crystal structure (PDB:1AMU; lightpink), and mapping of the active site residues of the crystal (Gly-324, Ile-330) onto d) asaC (Gly-301, Ile-307) and chyA’s (e) first (Gly-484, Gly-490) and f) second A domain (Ala-1571, Val-1577).

To assess the structural similarities of the active sites of the candidates, asaC and chyA, a crystal structure of a bacterial NRPS gramicidin synthase (GrsA) in complex with phenylalanine was used [23]. The complex with co-crystallized substrate allowed an identification of the active site residues that are in contact with the substrate. A superposition of a fungal crystal lacking a substrate (PDB:3ITE) to the bacterial crystal indicated that the active sites of the crystals were highly similar to each other (Suppl. Fig. 1). Thus, the active site residues in contact with the substrate in asaC and in chyA were inferred via mapping from GrsA (Ile330, Gly234) to the asaC AlphaFold protein structure model (Ile307, Gly301; Fig. 4 B, D) and chyA (Gly484, Gly490, Gly1571, Val1577; Fig. 4 C, E, F). These residues and the Stachelhaus code residues [24] were found in the active site of the GrsA crystal structure in close proximity to the substrate (Fig. 4 A). Since substitutions to amino acids with markedly different chemical characteristics at the active site may alter the binding affinity in NRPS [24], structurally similar proteins with an identical active site residue composition would be priority candidates. 24 candidate aspergillic acid synthetases had equal active site residue composition as asaC. Out of those 24, A0A077NGS5 and A0A01L9MVZ8 displayed the highest asaC sequence coverages of 95 % and 76 %, respectively (Suppl. Table 1, 3; Fig. 3 A). However, none of the chrysogine synthetase candidates displayed an active site composition identical to chyA. E1ACQ0 was the closest match differing by a deletion of Gly1571 (Suppl. Table 2, 4; Fig. 3 B). The lack of identical active site residue compositions to chyA was likely due to its more complex architecture compared to asaC, as well as the limited number of public sequence databases we queried for the demonstration purpose.

## 4 Conclusions

Our results showed that the sequence similarity searches can enrich both structurally similar and dissimilar proteins. Hence, filtering of enzyme candidates by structural similarity to the seed is necessary for identifying those that are the most likely to share the seed’s primary function. Filtering by structural similarity appeared particularly relevant when the NRPS enzyme candidates fell into distinct phylogenetic clusters and had higher sequence variance (Suppl. Fig. 2). Sufficient sequence variance is important for the likelihood of finding enzymes with different performance characteristics. Thus, our method allowed finding candidate enzymes having sequence variance but similar global folds and key active site residues as the seed enzyme. The filtering by structural similarity could refine NRPS candidate sets independently of the sequence resource and also when diverse, instead of equal, functionalities are searched from e.g., environmental DNA resources [25]. Refined enzyme candidate sets encourage for experimental screening also when the experimental capacity and resources are limited.

## CRediT authorship contribution statement

**Paula Jouhten:** Writing – review & editing, Supervision, Funding acquisition, Conceptualization. **Sandra Castillo:** Writing – review & editing, Supervision, Methodology, Conceptualization. **Linda Porri:** Writing – review & editing, Writing – original draft, Visualization, Methodology, Investigation, Formal analysis.

## Declaration of Competing Interest

The authors declare the following financial interests/personal relationships which may be considered as potential competing interests: Paula Jouhten reports financial support was provided by Research Council of Finland. Paula Jouhten reports financial support was provided by Novo Nordisk Foundation. If there are other authors, they declare that they have no known competing financial interests or personal relationships that could have appeared to influence the work reported in this paper.

## Footnotes

Appendix A Supplementary data associated with this article can be found in the online version at doi:10.1016/j.csbj.2025.09.039.
