# Supplementary material for: Structure-filtered search of enzyme variants
Source: Comput Struct Biotechnol J. 2025 Oct 1;27:4226–31. doi: 10.1016/j.csbj.2025.09.039 (PMC12513185; doi:10.1016/j.csbj.2025.09.039)
Supplement: Supplementary file 1 — Supplementary material [file mmc1.docx]

**Suppl. Fig. 1:** a) Global superposition of the bacterial A domain crystal complexed with phenylalanine (PDB:1AMU; lightpink) to the fungal A domain crystal lacking a co-crystallized substrate (PDB:3ITE; green). b) Active site residues as identified earlier in GrsA (PDB:1AMU; magenta) were mapped onto the fungal crystal (PDB:3ITE; darkgreen).

**Suppl. Fig. 2:** Evolutionary relationships of enzyme variants. Hierarchical clustering and active site variability of the structure-filtered enzyme candidates for a) asaC (B8N0E8) and b) chyA (B6HLP9).

**Acknowledgments**

Paula Jouhten acknowledges funding from Research Council of Finland (decision number 352417) and Novo Nordisk Foundation (NNF22OC0080180).

**References**

1. Huang B, Guo J, Yi B, Yu X, Sun L, Chen W. Heterologous production of secondary metabolites as pharmaceuticals in Saccharomyces cerevisiae. Biotechnol Lett. 2008 July;30(7):1121–37.

2. Victorino Da Silva Amatto I, Gonsales Da Rosa‐Garzon N, Antônio De Oliveira Simões F, Santiago F, Pereira Da Silva Leite N, Raspante Martins J, et al. Enzyme engineering and its industrial applications. Biotechnol Appl Biochem. 2022 Apr;69(2):389–409.

3. Bloom J, Meyer M, Meinhold P, Otey C, Macmillan D, Arnold F. Evolving strategies for enzyme engineering. Curr Opin Struct Biol. 2005 Aug;15(4):447–52.

4. Baker M. Protein engineering: navigating between chance and reason. Nat Methods. 2011 Aug;8(8):623–6.

5. Pearson WR. Selecting the Right Similarity‐Scoring Matrix. Curr Protoc Bioinforma [Internet]. 2013 Oct [cited 2025 July 18];43(1). Available from: https://currentprotocols.onlinelibrary.wiley.com/doi/10.1002/0471250953.bi0305s43

6. Caboche S, Leclère V, Pupin M, Kucherov G, Jacques P. Diversity of Monomers in Nonribosomal Peptides: towards the Prediction of Origin and Biological Activity. J Bacteriol. 2010 Oct;192(19):5143–50.

7. Dell M, Dunbar KL, Hertweck C. Ribosome-independent peptide biosynthesis: the challenge of a unifying nomenclature. Nat Prod Rep. 2022;39(3):453–9.

8. Süssmuth RD, Mainz A. Nonribosomal Peptide Synthesis—Principles and Prospects. Angew Chem Int Ed. 2017 Mar 27;56(14):3770–821.

9. Kaniusaite M, Goode RJA, Tailhades J, Schittenhelm RB, Cryle MJ. Exploring modular reengineering strategies to redesign the teicoplanin non-ribosomal peptide synthetase. Chem Sci. 2020;11(35):9443–58.

10. Brown AS, Calcott MJ, Owen JG, Ackerley DF. Structural, functional and evolutionary perspectives on effective re-engineering of non-ribosomal peptide synthetase assembly lines. Nat Prod Rep. 2018;35(11):1210–28.

11. Baunach M, Chowdhury S, Stallforth P, Dittmann E. The Landscape of Recombination Events That Create Nonribosomal Peptide Diversity. Barlow M, editor. Mol Biol Evol. 2021 May 4;38(5):2116–30.

12. Challis GL, Ravel J, Townsend CA. Predictive, structure-based model of amino acid recognition by nonribosomal peptide synthetase adenylation domains. Chem Biol. 2000 Mar;7(3):211–24.

13. Altschul S. Gapped BLAST and PSI-BLAST: a new generation of protein database search programs. Nucleic Acids Res. 1997 Sept 1;25(17):3389–402.

14. Li W, Godzik A. Cd-hit: a fast program for clustering and comparing large sets of protein or nucleotide sequences. Bioinformatics. 2006 July 1;22(13):1658–9.

15. Thompson JD, Higgins DG, Gibson TJ. CLUSTAL W: improving the sensitivity of progressive multiple sequence alignment through sequence weighting, position-specific gap penalties and weight matrix choice. Nucleic Acids Res. 1994 Nov 11;22(22):4673–80.

16. Letunic I, Bork P. Interactive Tree Of Life (iTOL) v5: an online tool for phylogenetic tree display and annotation. Nucleic Acids Res. 2021 July 2;49(W1):W293–6.

17. Zhang Y. TM-align: a protein structure alignment algorithm based on the TM-score. Nucleic Acids Res. 2005 Apr 11;33(7):2302–9.

18. Cock PJA, Antao T, Chang JT, Chapman BA, Cox CJ, Dalke A, et al. Biopython: freely available Python tools for computational molecular biology and bioinformatics. Bioinformatics. 2009 June 1;25(11):1422–3.

19. Dutcher JD. Aspergillic acid; an antibiotic substance produced by Aspergillus flavus. J Biol Chem. 1958 June;232(2):785–95.

20. Lebar MD, Cary JW, Majumdar R, Carter-Wientjes CH, Mack BM, Wei Q, et al. Identification and functional analysis of the aspergillic acid gene cluster in Aspergillus flavus. Fungal Genet Biol. 2018 July;116:14–23.

21. Viggiano A, Salo O, Ali H, Szymanski W, Lankhorst PP, Nygård Y, et al. Pathway for the Biosynthesis of the Pigment Chrysogine by Penicillium chrysogenum. Parales RE, editor. Appl Environ Microbiol [Internet]. 2018 Feb 15 [cited 2025 July 18];84(4). Available from: https://journals.asm.org/doi/10.1128/AEM.02246-17

22. Lannelongue L, Inouye M. Environmental Impacts of Machine Learning Applications in Protein Science. Cold Spring Harb Perspect Biol. 2023 Dec;15(12):a041473.

23. Conti E. Structural basis for the activation of phenylalanine in the non-ribosomal biosynthesis of gramicidin S. EMBO J. 1997 July 15;16(14):4174–83.

24. Stachelhaus T, Mootz HD, Marahiel MA. The specificity-conferring code of adenylation domains in nonribosomal peptide synthetases. Chem Biol. 1999 Aug;6(8):493–505.

25. Wu C, Shang Z, Lemetre C, Ternei MA, Brady SF. Cadasides, Calcium-Dependent Acidic Lipopeptides from the Soil Metagenome That Are Active against Multidrug-Resistant Bacteria. J Am Chem Soc. 2019 Mar 6;141(9):3910–9.
