# Supplementary figures and images for: Structure-filtered search of enzyme variants
Source: Comput Struct Biotechnol J. 2025 Oct 1;27:4226–31. doi: 10.1016/j.csbj.2025.09.039 (PMC12513185; doi:10.1016/j.csbj.2025.09.039)

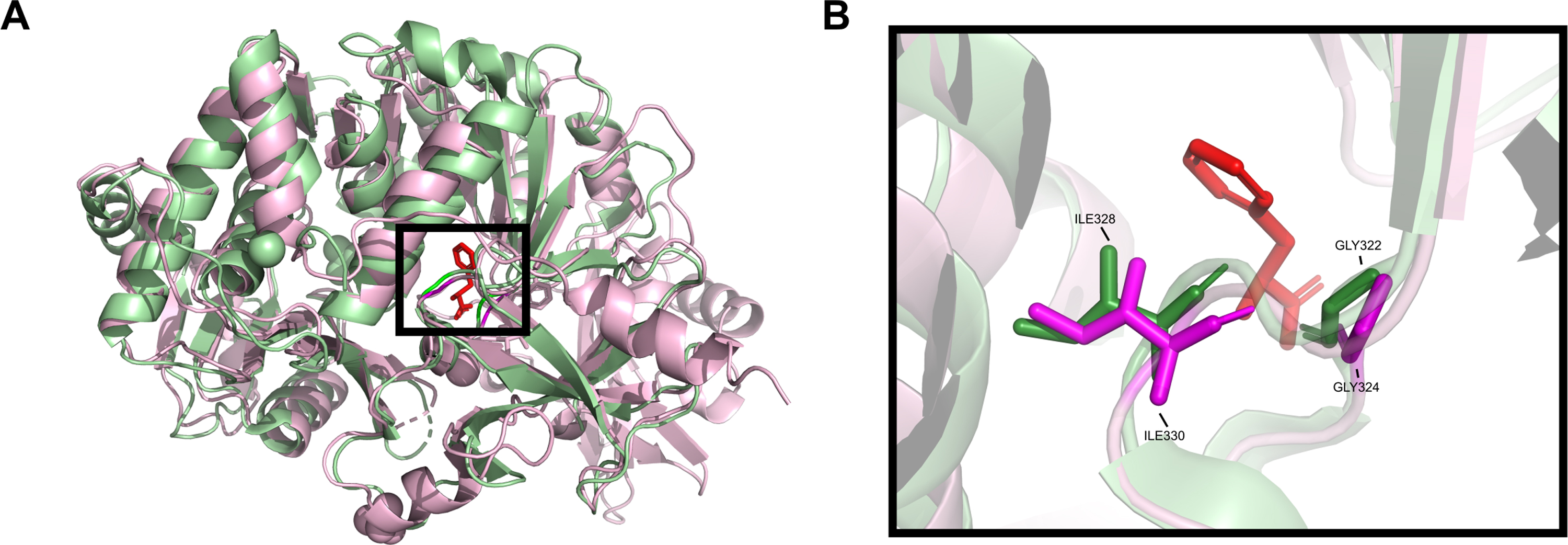

Supplement: Supplementary file 3 — Supplementary material [file mmc3.jpg]

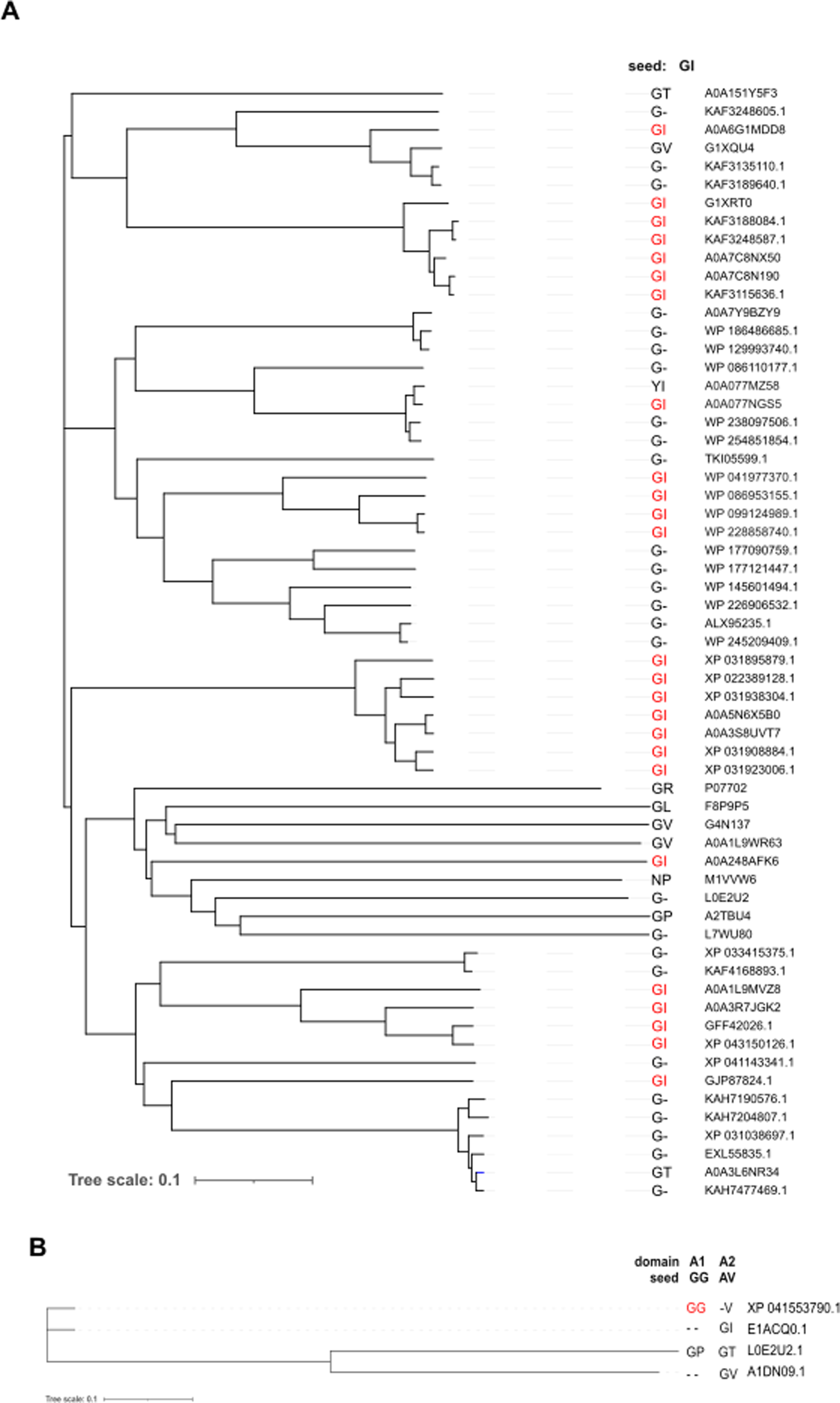

Supplement: Supplementary file 4 — Supplementary material [file mmc4.jpg]
